# Ammonium supply represses iron limitation to support Symbiodiniaceae growth

**DOI:** 10.3389/fmicb.2025.1663314

**Published:** 2025-10-28

**Authors:** Joshua James N. Versola, Hannah G. Reich, Irene B. Rodriguez

**Affiliations:** ^1^The Marine Science Institute, College of Science, University of the Philippines Diliman, Quezon City, Philippines; ^2^Bolinao Marine Laboratory, UP Marine Science Institute, Bolinao, Pangasinan, Philippines; ^3^Department of Biology, SUNY College of Environmental Science and Forestry Syracuse, New York, NY, United States

**Keywords:** trace metals, nutrients, macromolecules, pigments, algal symbionts, microalgae

## Abstract

Nutrient exchanges promote the success of symbioses among reef-building corals, endosymbiotic dinoflagellates (Family: Symbiodiniaceae), and their microbial symbionts. Nutrient dynamics has considerable implications on the metabolism and proliferation of the coral holobiont, with nutrient limitation known to increase the susceptibility of corals to bleaching by disrupting the host-symbiont nutrient exchange. This study examines how two Symbiodiniaceae species, *Symbiodinium microadriaticum* RT 362 and *Cladocopium goreaui* RT 152, respond to varying iron (Fe) availability, nitrate (NO_3_^−^), and ammonium (NH_4_^+^) in batch cultures. Under Fe limitation, phytoplankton growth is reduced when relying on NO₃^−^ due to the higher Fe requirement for nitrate assimilation enzymes, whereas NH_4_^+^ uptake is more efficient as it bypasses these Fe-dependent processes. Symbiodiniaceae utilize these nitrogenous compounds to fuel their metabolic processes, with an advantage in using NH_4_^+^ due to its greater energy efficiency and lower Fe requirement. Due to its role as cofactor of enzymes, Fe is crucial for nitrate reduction and chlorophyll synthesis, NH_4_^+^ assimilation remains effective even under low Fe conditions. The study reveals that in *S. microadriaticum* and *C. goreaui*, chlorophyll production, closely linked to Fe availability, and significantly influences carbohydrate and lipid synthesis, with both species boosting protein and carotenoid production under low Fe conditions. Chlorophyll and the other photosynthetic macromolecule product concentrations continue to increase with NH_4_^+^ as the N source, even under low Fe conditions. These findings offer critical insights into how these species adapt to varying environmental conditions, improving our understanding of coral resilience.

## Introduction

Symbiodiniaceae comprise a family of dinoflagellates that are key algal symbionts of many corals and other cnidarians, which are keystone organisms in coral reef ecosystems (Rädecker et al., 2015). Symbiodiniaceae species utilize carbon and nitrogenous compounds from the host in exchange for protection to produce photosynthates and facilitate uptake of dissolved inorganic nutrients within the coral holobiont (Grover et al., 2003; Morris et al., 2019). The loss of Symbiodiniaceae due to thermal and light stress, a phenomenon known as bleaching, is a concern because it disrupts the symbiotic relationship which is an anchor in the resilience of coral reefs. The increased availability of dissolved inorganic nitrogen (DIN), including nitrate (NO_3_^−^) and ammonium (NH_4_^+^), influences thermal tolerance in Symbiodiniaceae and these different DIN forms were shown to have varying effects on bleaching events in corals (Hoegh-Guldberg et al., 2007). Nitrate reduction and ammonium assimilation are two distinct nitrogen (N) metabolic pathways with key chemical differences and energy requirements. Nitrate reduction is a preparatory process that converts oxidized N into a bioavailable reduced form, whereas ammonium assimilation integrates N directly into amino acids for cellular metabolism (Glibert et al., 2016). In corals, the abundant supply of NH_4_^+^ can stimulate photosynthesis and can lessen effects of thermal stress by favoring the oxidative status and energy metabolism of the coral holobiont, helping maintain photoprotective pigmentation and delay bleaching (Fernandes de Barros Marangoni et al., 2020; Morris et al., 2019). In contrast, high NO_3_^−^ levels exacerbate bleaching under thermal stress (Burkepile et al., 2020; Wiedenmann et al., 2013). Although both nitrogenous compounds affect Symbiodiniaceae growth, the impacts of different DIN forms on photosynthetic activities vary, thus influencing the overall symbiosis differently (Li et al., 2021).

The availability of metals such as iron (Fe), zinc (Zn), manganese (Mn), and others, have significant biochemical impacts influencing growth and productivity in phytoplankton. In photosynthesis, Fe-containing cytochromes and Fe-sulfur clusters mediate in electron transport, while Mn is essential for the oxygen-evolving complex of photosystem II, driving light harvesting and water-splitting reactions. In nitrogen assimilation, Fe serves as a cofactor in nitrate and nitrite reductase, enabling the reduction of oxidized N forms (Mills et al., 2004; Schoffman et al., 2016). Antioxidant defense also relies on these metals: Mn and Fe act as cofactors in Mn- and Fe-superoxide dismutases (SODs), while Cu and Zn function in Cu/Zn-SOD, collectively detoxifying reactive oxygen species (ROS) (Alejandro et al., 2020; Mohandass et al., 2021; Schmidt and Husted, 2019; Zhang et al., 2008). For carbon fixation, Zn plays a critical role in carbonic anhydrase, which accelerates CO_2_-bicarbonate interconversion and facilitates photosynthetic carbon assimilation, in addition to stabilizing protein structures such as Zn-finger transcription factors (Coleman, 1992; McCall et al., 2000). Aside from these, Co, the central atom in vitamin B_12_ (also known as cobalamin), is required for DNA synthesis, methylation, and fatty acid metabolism, while Cu contributes to respiration through cytochrome c oxidase in oxidative phosphorylation. These metals are requisite for metabolic and physiological processes, and their precise concentrations must be tightly regulated, as deficiencies or excess can disrupt phytoplankton cellular function, highlighting their indispensable role in sustaining microalgae life (Manic et al., 2024; Peters et al., 2024).

The effects of varying DIN forms on Symbiodiniaceae is further aggravated by Fe limitation (Maldonado and Price, 1999; Sunda and Huntsman, 1997). Iron is indispensable due to its flexible redox chemistry that is crucial for many metabolic pathways including those involving macronutrients like N, phosphorus (P), and silicates (Si) (Morel and Price, 2003; Schoffman et al., 2016). Studies show that Fe requirements of phytoplankton vary with the available N species (Morel et al., 1991; Price et al., 1991; Wang and Dei, 2001). Under Fe limitation with NO_3_^−^ as the N source, acquisition and assimilation are less efficient, resulting in limited cell growth (Mills et al., 2004; Schoffman et al., 2016). Phytoplankton growth that depends on NO_3_^−^ requires additional Fe for NO_3_^−^ assimilation, particularly for enzymes like nitrate and nitrite reductases while those that rely on NH_4_^+^ as the N source demands less Fe as it can be directly incorporated into the cell in amino acids (Raven, 1988). Symbiodiniaceae may utilize both N forms, NO_3_^−^ and NH_4_^+^, in a balanced manner. In previous studies, Symbiodiniaceae showed a preference for NH_4_^+^ over NO_₃_^−^ (Li et al., 2021). This preference for NH_4_^+^ is associated with the lower energy cost on assimilation of NH_4_^+^ compared to NO_3_^−^, which requires multiple enzymatic reductions consuming ATP and NADPH (Miller and Cramer, 2005). The rapid assimilation of NH_4_^+^ into biomass is facilitated by the enzymes glutamine synthetase and glutamate synthase in Symbiodiniaceae, which also causes the activity of nitrate reductase and nitrite reductase to be down-regulated (Glibert et al., 2016; Miller and Cramer, 2005; Sanz-Luque et al., 2015).

In hospite, the availability of Fe and N for Symbiodiniaceae in corals is primarily regulated by the host rather than by abiotic factors. The host coral controls the supply of these essential nutrients through mechanisms such as the production of mucus, which can influence nutrient delivery to the symbionts, and the regulation of cellular processes that affect nutrient uptake and distribution (Cui et al., 2022; Pupier et al., 2021). This host-mediated regulation of Fe and N availability not only supplies Symbiodiniaceae with the nutrients required for metabolism but also constrains symbiont proliferation, thus maintaining a stable partnership despite fluctuating environmental conditions (Morris et al., 2019). In contrast to their symbiotic state, free-living Symbiodiniaceae must acquire Fe and N directly from the surrounding water. Their nutrient availability is therefore directly linked to abiotic factors and fluctuates with environmental conditions. Symbiodiniaceae can utilize different strategies to acquire nutrients, such as producing siderophores to scavenge for Fe or using specific transporters to absorb DIN compounds (Dellisanti et al., 2025). The contrasting nutrient acquisition strategies between symbiotic and free-living Symbiodiniaceae highlight the fundamental difference in their ecophysiology. As key primary producers, Symbiodiniaceae plays a crucial role in marine ecosystems, forming the base of the food web and supporting higher trophic levels. Symbiodiniaceae produces photosynthates such as glucose and amino acids, which are vital for corals and other invertebrates (Martinez et al., 2022; Muscatine and Porter, 1977). In addition, as free-living organisms, Symbiodiniaceae continue to produce these macromolecules, supporting their own growth and providing potential nutrient sources in the surrounding environment (Bhattacharya et al., 2024). Thus, their response to nutrient availability shapes productivity, community composition, and biogeochemical cycles, highlighting the need to understand their adaptation strategies.

This study focused on two species, *Symbiodinium microadriaticum* and *Cladocopium goreaui*, that are widely spread among different coral species across tropical and subtropical waters globally (Baker, 2003; LaJeunesse, 2005; LaJeunesse et al., 2018). Strains of *S. microadriaticum* can confer thermal resilience to their coral hosts, enhancing the ability of the coral holobiont to withstand elevated water temperatures. This trait is critical for coral survival during climate change-induced bleaching events (Howells et al., 2012). Studies on *C. goreaui* have focused on its adaptive capabilities to different environmental conditions including temperature stress (Baird et al., 2007; LaJeunesse et al., 2018). These studies revealed that certain strains of *C. goreaui* may exhibit different thermal tolerances compared to *S. microadriaticum*, suggesting a complex interplay between these symbionts and their coral hosts in response to environmental changes (Baird et al., 2007; Silverstein et al., 2012). While both species fulfill similar ecological roles as photosynthetic symbionts, they may exhibit distinct physiological responses to environmental conditions, contributing to the overall resilience of the coral holobiont. Thus, we wanted to contribute to exploring variabilities among species and understanding the mechanisms underlying algal-host symbioses and their implications for reef health and conservation. Our study aimed to address the limited knowledge of how Fe availability and nitrogenous compounds affect the growth, nutrient utilization, and biochemical composition of two Symbiodiniaceae species. Reducing the availability of Fe will differentially affect the utilization of either NO_3_^−^ and/or NH_4_^+^, and may affect growth, photosynthetic performance, and biochemical composition of *S. microadriaticum* and *C. goreaui*. By exploring their specific responses to varying Fe and N conditions, we seek to gain insights into their fitness and resilience in changing marine ecosystem.

## Materials and methods

### Batch culture conditions and growth monitoring

**Figure 1:**
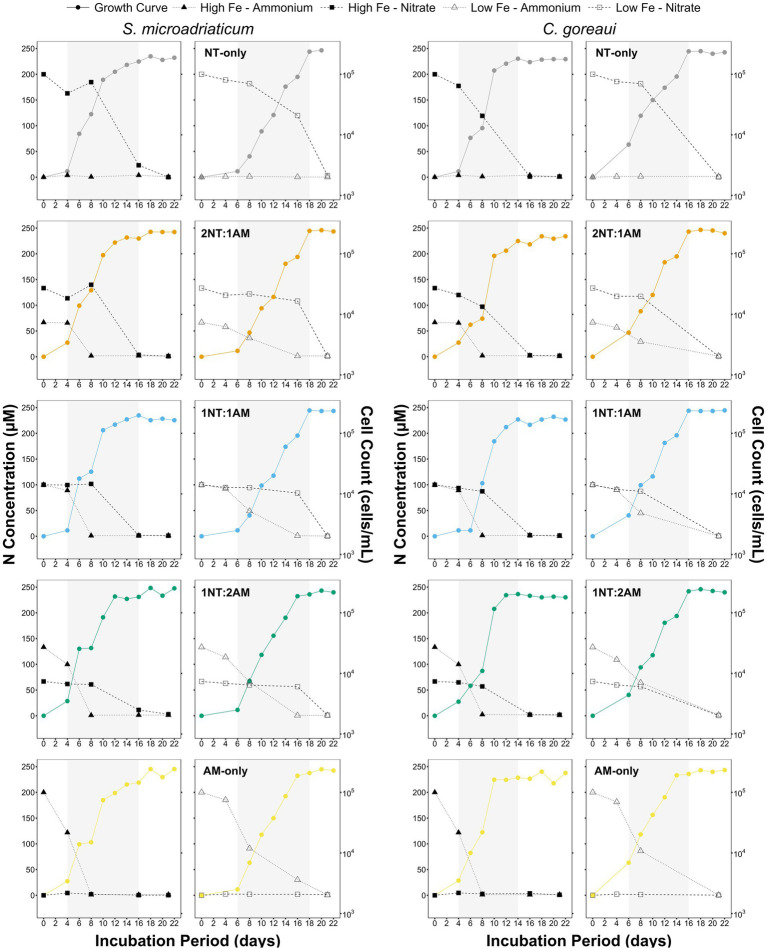
Concentration of NO_3_^−^ and NH_4_^+^ in medium and growth curves of *S. microadriaticum* and *C. goreaui* subjected to varying Fe and N treatments. Points represent mean values from triplicate culture bottles. Shaded region represents exponential growth phase of *Symbiodiniaceae* per treatment.

Non-axenic cultures of *S. microadriaticum* RT 362 and *C. goreaui* RT 152 were acclimatized under different Fe-N conditions and grown in 500 mL polycarbonate bottles under a 12:12 light–dark cycle of light supplied at 390 ± 10 μmol photons m^−2^ s^−1^ and temperature kept at 28 °C ± 2 °C. The trace-metal defined medium was patterned from the modified L1 recipe (Supplementary Table S1) (Rodriguez et al., 2016), prepared using filter-sterilized (0.22 μm Stericap^™^) seawater from Bolinao, Pangasinan, based on the original recipe of (Guillard and Hargraves, 1993; Rodriguez et al., 2016). High (total dissolved concentration of 100 nM Fe, HFe from here onwards) and low (total dissolved concentration of 20 nM Fe, LFe from here onwards) Fe treatments were supplemented with one of five different N ratios: NO_3_^−^ only (NT only), NH_4_^+^ only (AM only), and three mixed ratios (2NT:1AM, 1NT:1AM, and 1NT:2AM) at a total N concentration of 200 μM, yielding 10 experimental treatments with three individual biological replicates (bottles) per species. These concentrations were selected to ensure that cultures could reach sufficient cell densities for comparative analyses of macromolecules and intracellular metal quotas while still eliciting physiologically relevant responses. Growth and cell density changes were monitored every other day, for 22 days, using a hemocytometer. Specific growth rates (μ) were calculated using the equation: μ = ln (N_x_−N_o_)/(T_x_−T_o_), where T_o_ is the start day and T_x_ is the end day of the exponential phase (Reich et al., 2020). The exponential phase, characterized by maximal and uninhibited growth where cell numbers double at a constant rate, ends as the growth slows and eventually stops upon reaching maximum cell density (Figure 1).

### Determination of available nitrogen in medium during algal growth

The nitrogen consumed by algal cells during growth was indirectly assessed by measuring the NO_3_^−^ and NH_4_^+^ concentrations in growth media. Samples were collected at five growth stages: initial (day 0), pre-exponential, mid-exponential, post-exponential, and stationary phases. Approximately 12 mL of each sample was filtered using a 0.45 μm cellulose acetate filter. Concentrations of NO_x_ (NO_3_^−^ + NO_2_^−^) and NH_4_^+^ in the filtered spent medium were determined through spectrophotometric analysis using a nutrient analyzer (San++^®^ Series - Automated Wet Chemistry Analyzer, Skalar). The nutrient analyzer measures NO_x_ by first reducing nitrate to nitrite in a cadmium–copper column, then applying the Griess reaction to produce a pink azo dye, with absorbance measured at 540 nm (Niu et al., 2023). Ammonium is determined using the indophenol blue method, where it reacts with phenol and hypochlorite in the presence of sodium nitroprusside to form a blue complex measured at 630 nm (Aminot et al., 1997). Both analyses are performed in a continuous flow system, where samples and reagents are mixed and segmented with air bubbles to enhance mixing, minimize carryover, and enable precise photometric detection.

### Intracellular metal content or metal quotas

Separate samples of about 10 million Symbiodiniaceae cells were collected for each late exponential phases per treatment (day 16 for HFe treatments and day 18 for LFe treatments of *S. microadriaticum*, and; day 14 for HFe treatments and day 16 for LFe treatments of *C. goreaui*). Cells were harvested by filtration onto acid-washed 2 μm pore-sized polycarbonate filters, which were transferred to Teflon^®^ vials, washed with ultrapure water, and digested with concentrated trace metal grade nitric acid (HNO_3_) prior to dilution with ultrapure water (Rodriguez et al., 2016). Diluted samples were analyzed for trace metal content using a high resolution inductively coupled plasma mass spectrometer (Element 2^™^ HR-ICP-MS, Thermo Scientific^™^). The metal quotas were normalized against the phosphorus (P) content as biomass indicator and presented as mmol mol^−1^ P to capture cellular stoichiometry and facilitate comparisons across treatments, following (Reich et al., 2020; Rodriguez and Ho, 2018).

### Analysis of Symbiodiniaceae macromolecule and pigment content

Various macromolecule and pigment content of *S. microadriaticum* and *C. goreaui* cells were measured using spectrophotometric assays to assess the physiological state and metabolic health across different Fe and N treatments. About 10 million cells for pigments and 6 million cells for other macromolecules were also collected during the late exponential phase and pelletized by centrifugation at 6,000 rpm for 15 min. The pellets were resuspended in 0.1 M phosphate buffer (prepared at pH ~ 7.4) prior to respective spectrophotometric assays using a UV–Vis spectrophotometer (UV-1900i, Shimadzu). Cell lysis was carried out by sonication at 100% amplitude setting using two 30-s short pulses, separated by a 15-s rest interval (Q55 Sonicator 20 kHz, QSonica). The total carbohydrate content was determined on cell pellets treated with 75% sulfuric acid and Anthrone solution (Morris, 1948). The carbohydrate content was quantified by measuring absorbance at 578 nm. For protein quantification, cells lysed were precipitated with 10% trichloroacetic acid (TCA) and pelletized using centrifugation. Protein pellets were then resuspended in phosphate-buffered saline, mixed with Bradford reagent, and absorbance was measured at 595 nm (Bradford, 1976). Lipid content was assessed by extracting cells with a 2:1 chloroform-methanol mixture. The separated organic layer was treated with concentrated sulfuric acid and phospho-vanillin reagent prior to absorbance measurements at 530 nm (Park et al., 2016). Pigments, including chlorophyll and carotenoids, were extracted with 80% cold acetone and concentrations were determined by measuring absorbance at 663, 645, and 470 nm (Lichtenthaler and Wellburn, 1987). All macromolecules and pigments were reported as weight per cell of Symbiodiniaceae (pg cell^−1^).

### Statistical analyses

All statistical analyses were conducted in R version 4.5.0. The Shapiro–Wilk test checked normality and Levene’s test assessed homogeneity of variance for each variable. A three-way analysis of variance (ANOVA) was conducted to assess significant differences between species, Fe level and N ratio, followed by Tukey’s Honestly Significant Difference (TukeyHSD) test to compare data among all treatments and a Bonferroni correction for *post-hoc* pairwise t test comparisons per treatment between species of Symbiodiniaceae. Principal component analysis (PCA) was performed using the *FactoMineR* and *factoextra* packages to reduce dimensionality and visualize data structure. All results are expressed as mean ± standard deviation unless stated otherwise.

## Results

The growth rates of *S. microadriaticum* and *C. goreaui* varied distinctly under different Fe and N conditions (Table 1). HFe conditions reflect species-specific physiological responses to nutrient availability (Supplementary Table S3). Across all treatments, Fe availability played a critical role in influencing growth rates, with both species exhibiting significantly higher growth rates under HFe conditions compared to LFe conditions (*p* < 2 × 10^−16^, 3-way ANOVA). In *S. microadriaticum*, the highest growth rates were observed in HFe conditions, confirming the positive role of Fe in supporting cellular growth (Table 1). Under LFe conditions, growth rates were generally lower, indicating the physiological constraints imposed by decreased Fe availability. However, within LFe conditions, N availability influenced growth, with the AM-only (0.31 ± 0.008) and the 1NT:2AM. (0.31 ± 0.007) treatments supporting significantly higher growth rates. Notably, under NH_4_^+^-rich conditions, growth rates in LFe conditions were comparable to those observed in HFe conditions, suggesting that NH_4_^+^ supplementation partially alleviated the effects of Fe limitation in *S. microadriaticum*. Similarly, *C. goreaui* exhibited higher growth rates in HFe conditions compared to LFe conditions, highlighting the importance of Fe in sustaining cellular growth (Table 1). Decreasing Fe had similar impact on both species as evidenced by no significant differences found in the growth rates under LFe conditions (Supplementary Table S3) Among the N treatments, growth rates under LFe conditions were highest in AM-only treatments (0.32 ± 0.005), indicating that NH_4_^+^ supported growth under low Fe availability. However, unlike *S. microadriaticum*, where certain N treatments in LFe conditions resulted in growth rates on par with HFe conditions, *C. goreaui* still exhibited significantly lower growth rates in LFe conditions compared to HFe, even under abundance of NH_4_^+^ (Table 1). This suggests that while NH_4_^+^ partially mitigated the limited Fe available, *C. goreaui* remained more constrained by low Fe availability than *S. microadriaticum*.

**Table 1:** Specific growth rates of *S. microadriaticum* and *C. goreaui* subjected to varying Fe and N availability.

Species	Fe level	N Ratio				
		NT-only	2NT:1AM	1NT:1AM	1NT:2AM	AM-only
*S. microadriaticum*	High	0.327 ± 0.002^de^	0.326 ± 0.011^d^	0.326 ± 0.011^d^	0.319 ± 0.009^de^	0.308 ± 0.011^defg^
	Low	0.291 ± 0.005^fg^	0.290 ± 0.004^g^	0.291 ± 0.010^fg^	0.321 ± 0.007^def^	0.310 ± 0.008^defg^
*C. goreaui*	High	0.400 ± 0.004^ab^	0.365 ± 0.007^c^	0.390 ± 0.003^b^	0.417 ± 0.002^a^	0.383 ± 0.003^bc^
	Low	0.301 ± 0.008^efg^	0.308 ± 0.002^defg^	0.306 ± 0.004^defg^	0.307 ± 0.009^defg^	0.324 ± 0.005^d^

Letters indicate Tukey’s *post-hoc* groupings for significant differences analyzed per species.

In terms of cell densities, no significant differences were observed in the maximum values achieved by *S. microadriaticum* and *C. goreaui*. However, a key distinction was noted in the time elapsed to reach these maximum densities, with LFe treatments causing a two-day delay compared to HFe treatments (Figure 1). This delay coincided with lowered N concentrations in the medium in parallel with increasing cell counts across all treatments (Figure 1). Both species demonstrated efficient absorption of NO_3_^−^ and NH_4_^+^, with NH_4_^+^ being rapidly depleted in all scenarios, indicating its preferential absorption as a DIN source (Figure 1). Despite equivalent initial N concentrations across treatments, variations in NH_4_^+^and NH_4_^+^ levels in the medium highlighted that reduced Fe availability impairs NO₃^−^ uptake, contributing to delayed growth under low Fe conditions.

**Figure 2:**
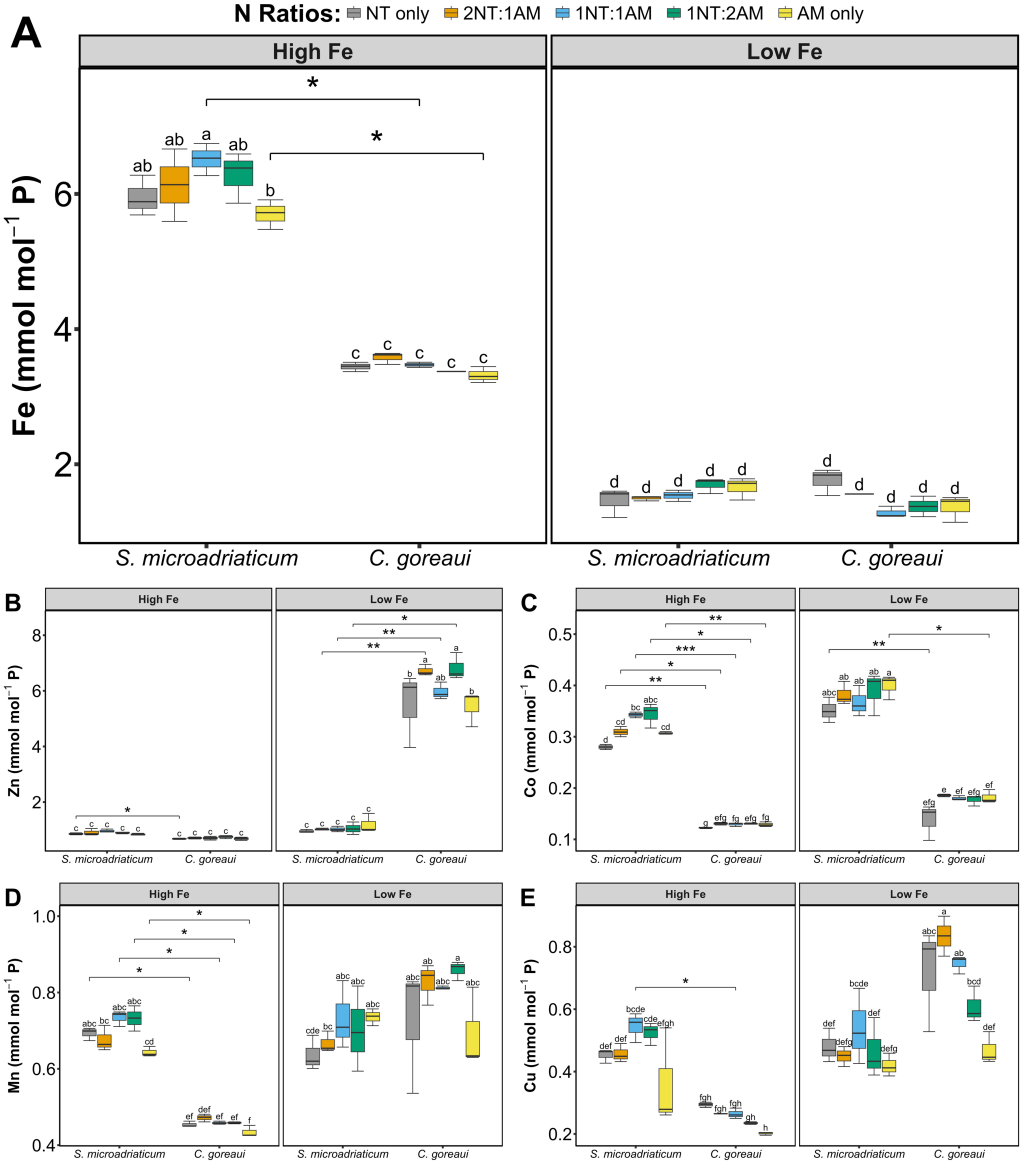
Intracellular Fe **(A)**, Zn **(B)**, Co **(C)**, Mn **(D)**, and Cu **(E)** concentration, normalized to phosphorus (mmol mol^−1^ P), of *S. microadriaticum* and *C. goreaui* subjected to varying Fe and N treatments. Boxplots represent range of values with error bars showing standard deviations from triplicate culture bottles. Letters above boxplots indicate Tukey’s *post-hoc* groupings for significant differences. Asterisks show significant differences between species on the same treatments.

Cell density and cellular P quotas exhibited contrasting but biologically coherent patterns across Fe and N treatments (Supplementary Figure S1). Under HFe, both *S. microadriaticum* and *C. goreaui* showed higher cell densities accompanied by slightly lower P per cell, consistent with P dilution during rapid proliferation. Conversely, under LFe, P quotas and cell densities remained relatively stable, reflecting slower growth and more uniform P allocation per cell. This inverse relationship between cellular P and growth rate represents an expected physiological adjustment in P metabolism under varying Fe and N availability. To correct for growth-related differences in biomass and cellular size, intracellular metal concentrations were normalized to P (mmol mol^−1^ P, Figure 2), with P serving as a proxy for total metabolic capacity. This normalization minimizes the effects of variable cell density and allows direct comparison of intracellular metal allocation across Fe and N treatments. Parallel analyses expressed on a per cell basis (mol cell^−1^, Supplementary Figure S2) produced similar overall trends, confirming that the P normalized data capture true physiological responses rather than artifacts of growth variability. Minor divergence between the two normalization approaches within specific N treatments reflects the expected inverse relationship between P per cell and cell density, further supporting the validity of P normalization for interpreting metal regulation in *S. microadriaticum* and *C. goreaui*.

In comparing the metal quotas of *S. microadriaticum* and *C. goreaui*, both species showed distinct patterns in response to metal availability, especially at HFe conditions. *Symbiodinium microadriaticum* exhibited a significant increase in Fe quotas under HFe conditions (*p* < 2 × 10^−16^, 3-way ANOVA), with minimal insignificant changes in Zn, Mn, and Cu (*p* < 2 × 10^−16^; *p* = 1.83 × 10^−13^; *p* = 1.96 × 10^−14^, respectively, 3-way ANOVA) (Figures 2A,B,D,E). Cobalt quotas show significant differences in *S. microadriaticum*, indicating changes in Co uptake as a response to varying Fe availability (*p* = 7.07 × 10^−14^, 3-way ANOVA) (Figure 2C). In contrast, while *C. goreaui* also showed a significant increase in Fe quotas under HFe conditions, the response was less pronounced than in *S. microadriaticum* (Figure 2A). However, other metal quotas (Zn, Co, Mn, Cu) in *C. goreaui* increased significantly under LFe conditions, indicating that this species compensated for low Fe availability by upregulating the uptake of other metals, a trend not observed in *S. microadriaticum* (Figures 2B–E). Overall, *C. goreaui* appeared to be more responsive in regulating other essential metal uptake in response to varying Fe availability.

**Figure 3:**
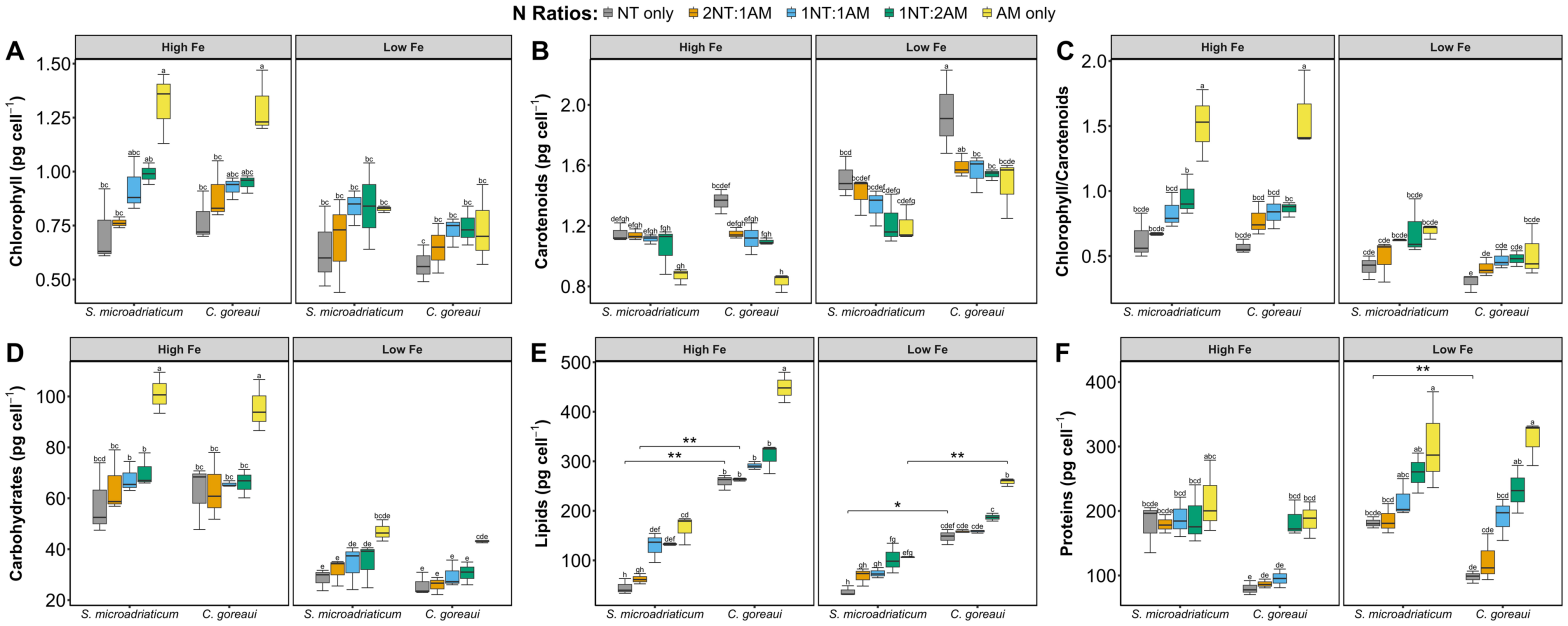
Chlorophyll **(A)**, carotenoid **(B)**, chlorophyll/carotenoid **(C)**, carbohydrate **(D)**, lipid **(E)**, and protein **(F)** contents, in pg cell^−1^, of *S. microadriaticum* and *C. goreaui* subjected to varying Fe and N treatments. Boxplots represent range of values with error bars showing standard deviations from triplicate culture bottles. Letters above boxplots indicate Tukey’s *post-hoc* groupings for significant differences. Asterisks show significant differences between species on the same treatments.

Macromolecules and pigments were strongly influenced by Fe and N. Chlorophyll content in both species was significantly higher in HFe conditions, particularly in AM-only treatments, which exhibited greater reductions under low Fe availability (*p* = 0.003, 3-way ANOVA) (Figure 3A). Carbohydrate accumulation as well as lipid concentrations had higher levels in HFe conditions (*p* < 2 × 10^−16^; *p* < 2 × 10^−16^, 3-way ANOVA) (Figures 3D,E). In contrast, protein levels were primarily influenced by N availability rather than Fe, with significantly higher protein content observed in NH_4_^+^-rich treatments (Fe level: *p* = 7.31 × 10^−7^ vs. N availability: *p* = 8.80 × 10^−11^; Fe-N effects: *p* = 0.013, 3-way ANOVA). The effect of N on protein accumulation was particularly pronounced in AM-only and 1NT:2AM treatments, reinforcing NH_4_^+^ as a more efficient N source for protein synthesis. In *S. microadriaticum*, AM-only treatments exhibited the highest protein levels. *Cladocopium goreaui* exhibited the same nitrogen-driven pattern, where NH_4_^+^-rich conditions were more pronounced than other *N* ratio treatments (Figure 3F).

Chlorophyll/carotenoids ratio in both species indicated a significant allocation of pigments for production of macromolecules in NH_4_^+^-rich conditions under HFe conditions, while an observed increasing allocation for photoprotection toward NO_3_^−^-rich conditions. Both species show carotenoid concentrations were higher under LFe conditions rather than in HFe conditions, with NO_3_^−^-rich conditions leading to increased carotenoid accumulation. The effect of N on carotenoid content was also dependent on the abundant N species, with *S. microadriaticum* and *C. goreaui* showing increasing carotenoid levels with increasing NO_3_^−^, specifically exhibiting the highest carotenoid levels under NO_3_^−^-rich treatments, NT-only and 2NT:1AM (Figure 3B).

**Figure 4:**
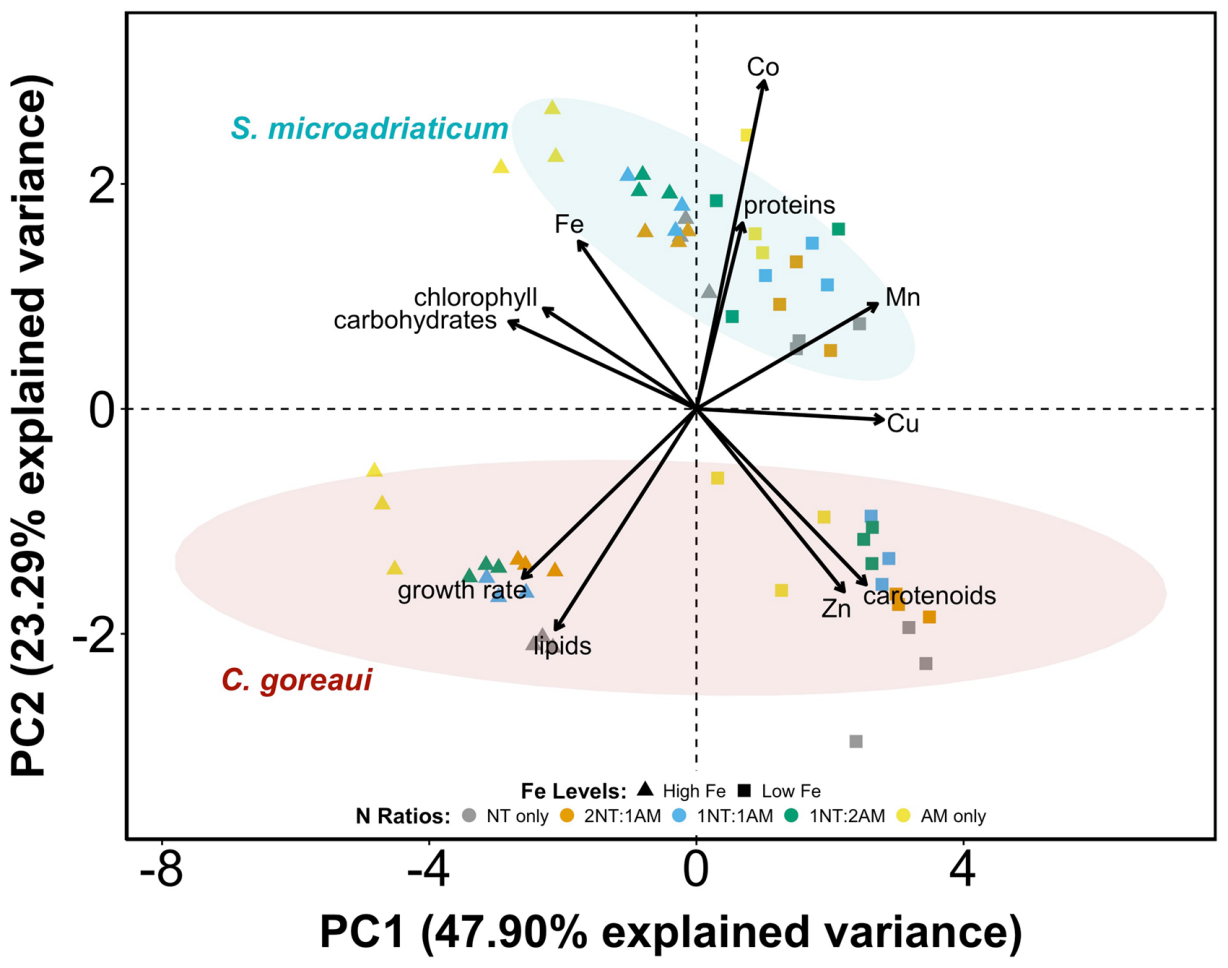
Principal component analysis (PCA) of macromolecules, pigments, and metal quotas per species of Symbiodiniaceae subjected to varying Fe and N treatments. Vector length is correlated with contribution in the first two principal components.

The PCA revealed distinct patterns of differentiation for *S. microadriaticum* and *C. goreaui* (Figure 4). The availability of Fe was the most significant driver of variance, primarily along PC1, which explained 47.90% of the variance. The separation of data points between HFe vs. LFe along PC1 highlights the relatively positive influence of Fe availability on parameters such as Fe quota, growth rate, chlorophyll, carbohydrates, and lipids in both species. It also showed the moderate influence of Fe on other metal quotas, proteins, and carotenoids. The influence of N source was seen along PC2, which explained 23.29% of the variance. The distribution of data points by N ratios, demonstrated that different N sources modulate macromolecules and pigments, although this effect was less pronounced than Fe availability. These variances reflected the unique physiological and metabolic responses of Symbiodiniaceae to Fe and N availability. Overall, Fe limitation emerged as the primary driver of variance in both species, followed by the influence of N source, with species-specific responses further contributing to the observed patterns.

## Discussion

The interplay between Fe and N availability is a critical factor dictating phytoplankton ecology and in shaping adaptive strategies employed under varying environmental conditions. In this work, we observed that growth of *S. microadriaticum* and *C. goreaui* was influenced by differing Fe and N availability. Our results show that lower Fe supply reduced growth rates of both *S. microadriaticum* and *C. goreaui* across all treatments, validating the important role of Fe in these species. Comparison between species supplied with either low Fe or high Fe showed that *S. microadriaticum* had lower specific growth rates compared with *C. goreaui* regardless of the N treatments (Table 1). This hints at the varying physiological requirements that may be linked to Fe utilization, which was reflected in Fe quotas displaying significant variations in *C. goreaui* and *S. microadriaticum* (Figure 2A). In the case of *S. microadriaticum*, the significant increase in Fe quotas under high Fe conditions suggests a tendency for luxury uptake and storage when Fe supply is abundant. However, the minimal changes in quotas of other essential metals, such as Zn, Mn, and Cu, imply that *S. microadriaticum* maintains a relatively stable metal homeostasis, prioritizing Fe acquisition over other metals (Figure 2). This could reflect a species-specific adaptation elicited by Fe requirement for metabolic functions, particularly those involved in photosynthesis and electron transport processes, without the need for compensatory changes in other metals. The results show that *C. goreaui* exhibited a more complex response to Fe availability. While Fe quotas increase under high Fe conditions, the response was less pronounced compared to *S. microadriaticum.* This suggests that *C. goreaui* may have different metabolic demands or tendency toward luxury uptake of Fe under high availability. However, a key difference is observed in its response to low Fe conditions, which showed higher metal quotas indicating enhanced uptake of other metals. This significant increase in intracellular content of other metals indicates that *C. goreaui* may have evolved a more flexible strategy to cope with Fe limitation by enhancing the acquisition of other essential metals that may fulfill some of the physiological roles typically reliant on Fe (Figure 2). For instance, Zn and Mn are cofactors in a variety of enzymatic reactions, and Co is important for vitamin B_12_ synthesis, a critical cofactor for certain metabolic pathways (Alejandro et al., 2020; Bohutskyi et al., 2019; Coleman, 1992; Cui et al., 2022; McCall et al., 2000; Rodriguez and Ho, 2015). Also, the significant difference in Cu quotas between species subjected to varying Fe conditions is noteworthy. Copper is essential for several enzymatic processes, including those involved in electron transport chains and photosynthesis. The Cu quota of many phytoplankton species is directly and physiologically linked to Fe availability (Peers et al., 2005). Under Fe-limited conditions, phytoplankton upregulate their Cu-dependent high-affinity Fe uptake systems, leading to an increased demand for Cu and a higher cellular Cu/C ratio. This physiological adaptation is a crucial survival strategy for these organisms in the vast, Fe-poor regions of the global ocean. The observation that Cu quotas remain unchanged in *S. microadriaticum* suggests that Cu homeostasis is tightly regulated and independent of Fe availability. This denotes that Cu acquisition and utilization are perhaps not directly influenced by Fe availability in this species, or that their Cu pools are maintained through a mechanism not strongly affected by Fe fluctuations. While a strong link exists between Fe limitation and increased Cu quotas for many phytoplankton species (especially oceanic diatoms), it is not a universal rule. The absence of this response can be attributed to the specific physiological adaptations of the species, the co-limitation by other nutrients, and the time-dependent nature of cellular acclimation.

The Fe and other metal requirements of these Symbiodiniaceae species may have been dictated by the ecological niches they occupy. Both *S. microadriaticum* and *C. goreaui* are widely distributed with the former occurring as a symbiotic, opportunistic, or free-living while the latter predominantly adopting a symbiotic lifestyle with specific hosts (Baker, 2003; LaJeunesse, 2005; LaJeunesse et al., 2018). The difference in lifestyles of free-living and symbiotic species may have shaped the metal requirements of these two species with the free-living able to develop the genetic machinery to acquire nutrients better. This warrants further studies using complementary approaches to better understand how free-living or symbiotic species may persist in challenging environments under limited supply of vital nutrients like Fe and N. One of the key functions of Fe in phytoplankton is anchored on the assimilation of different N sources, particularly as an essential cofactor of nitrate and nitrite reductases (Behrenfeld and Kolber, 1999; Sunda and Huntsman, 1997). Declining Fe availability can potentially decrease the activity of these enzymes and restrict the capacity of Symbiodiniaceae to make use of NO_3_^−^ efficiently. Adequate Fe ensures the proper function of Fe-dependent enzymes, while the form of N affects how efficiently Symbiodiniaceae can assimilate N and carry out subsequent metabolic processes (Geider and La Roche, 1994; Sunda and Huntsman, 1997).

Phosphorus quota is inversely proportional to cell density because, at higher population densities, individual cells experience reduced external P availability, leading to lower P quotas per cell as they conserve the limited nutrient to sustain division and maintain metabolic balance. As biomass accumulates rapidly under high Fe and N-abundant conditions, available P in the medium becomes quickly depleted, and cells adjust by reducing their intracellular P reserves relative to total biomass. To correct for this effect, intracellular metals were normalized to phosphorus (mmol mol^−1^ P, Figure 2), with P serving as an index of metabolic capacity. This approach reduces the influence of cell density and size variation, thereby allowing more direct comparison of intracellular metal allocation across Fe and N treatments. Phosphorus is tightly coupled to nucleic acids, ATP, and phospholipids, making P normalization a biologically meaningful reference point for metal homeostasis (Lin et al., 2016; Paytan and McLaughlin, 2007). Comparable P normalization effects have been reported in Symbiodiniaceae under different metal availability (Reich et al., 2020; Rodriguez and Ho, 2018), supporting the broader applicability of this normalization strategy for linking trace metal quotas to nutrient-driven metabolic states and providing a growth-independent perspective that bridges elemental stoichiometry with macromolecular and pigment composition.

Our results reveal patterns in chlorophyll, carbohydrate, and lipid contents that align closely with intracellular Fe content, highlighting the crucial role of Fe in modulating key metabolic processes in *S. microadriaticum* and *C. goreaui*. The strong influence of Fe on pigment synthesis, particularly chlorophyll, indicates that both species prioritize Fe for photosynthetic functions, which is essential for their energy production and overall metabolic health (Figure 3A). Iron is essential for chlorophyll production especially in nutrient-poor marine environments. Under limiting Fe conditions, chlorophyll production is impeded affecting photosynthetic activity and macromolecule production (Behrenfeld and Kolber, 1999; Boyd et al., 2007; Falkowski and Kolber, 1995). The observed patterns in chlorophyll content from varying N treatments underscore that growth conditions trigger nutrient-availability dependent adaptive responses. Chlorophyll converts light energy to produce ATP and NADPH, both of which are important factors for the synthesis of carbohydrates, thus a decrease in chlorophyll can reduce energy and resources for this metabolic process (Behrenfeld et al., 2006; Geider and La Roche, 1994). The observed increase in carbohydrate concentrations under high Fe conditions was a shared trend for both species, underscoring the role of Fe in promoting anabolic processes like carbon fixation (Figure 3D). This suggests that Fe availability enhances the metabolic activity and biosynthetic capacity of both species. For lipids, however, only *C. goreaui* showed an increase, while no changes were seen in *S. microadriaticum*, indicating *C. goreaui* was more significantly affected (Figure 3E). The higher accumulation of these key energy reserves and structural components under Fe-sufficient conditions reflects the enhanced metabolic activity and biosynthetic capacity of both species, which could also play a critical role in sustaining cellular energy balance and membrane integrity.

The determined protein levels show that under high Fe conditions, NO_3_^−^ dominant treatments have significantly lower protein accumulation than in NH_4_^+^ treatments (Figure 3F). This phenomenon has also been observed in previous studies where ammonium enrichment significantly boosts protein production in corals, highlighting the influence of specific N forms on the physiological processes within the coral-dinoflagellate symbiosis (Ezzat et al., 2015). Supporting this observation, ammonium assimilation via glutamine synthetase and glutamate dehydrogenase is energetically favorable and requires fewer enzymatic steps, leading to more efficient protein synthesis (Glibert et al., 2016; Miller and Cramer, 2005; Sanz-Luque et al., 2015). Conversely, nitrate assimilation demands higher energy input and involves iron-dependent enzymes, such as nitrate reductase, which may become limiting even under elevated Fe availability (Maldonado and Price, 1999; Sunda and Huntsman, 1997). The pronounced nitrogen-driven effect on protein accumulation in *C. goreaui* compared to *S. microadriaticum* suggests a species-specific difference in how nitrogen is assimilated and utilized for growth. *C. goreaui* appears to exhibit a more responsive or flexible nitrogen metabolism, which may be a strategy to maximize growth and survival under varying nitrogen conditions. This is supported by the greater differences in protein content between treatments in *C. goreaui* than in *S. microadriaticum*, further highlighting its sensitivity to nitrogen availability. These observations show the complex interplay between Fe availability, nitrogen source, and protein synthesis in Symbiodiniaceae, highlighting their adaptive responses to nutrient variability.

The carotenoid content in both study organisms increased with lower Fe levels across most N treatments (Figure 3B). The reduction is more pronounced in *C. goreaui*, suggesting a stronger downregulation of carotenoid biosynthesis pathways in elevated Fe levels. Carotenoids act as antioxidants, which neutralize ROS generated during photosynthesis and other metabolic processes, protecting the photosynthetic systems from oxidative damage (Maoka, 2020; Niyogi, 1999). When Fe levels are low, the high carotenoid production represents a protective mechanism to maintain photosynthetic efficiency (Maoka, 2020; Niyogi, 1999). Decreased Fe levels compromise the efficiency of the electron transport chain in chloroplasts because Fe is crucial for cytochromes and Fe-S proteins (Kroh and Pilon, 2020; López-Millán et al., 2016). Moreover, Fe reduction and higher levels of NO_3_^−^ can increase oxidative stress, making carotenoids essential as Symbiodiniaceae’s photoprotective pigment response for oxidative stress rather than in chlorophyll production for macromolecule production (Figure 3C). Decreasing Fe concentrations trigger adaptive strategies such as the production of Fe-independent proteins, which help maintain metabolic functions and photosynthetic efficiency. Interestingly, while there are a lot of metalloenzymes that play critical roles in antioxidative mechanisms, the PCA results show that proteins and carotenoids are not correlated with each other (Figure 4). Moreover, the loadings plot demonstrates that carotenoid production is not associated with the production of carbohydrates or lipids. This points to biological functions of carotenoids, other than antioxidative defense, that warrants further study.

The responses of *S. microadriaticum* and *C. goreaui* revealed both shared and divergent strategies in coping with Fe and N availability. In both species, Fe emerged as the dominant driver of physiological variance, with limitation consistently reducing growth and Fe quotas (Figure 4). *S. microadriaticum* exhibited modest changes under high Fe, maintaining relatively stable pigment levels and showing only slight lipid increases, consistent with a strategy that prioritizes photosynthesis over carbon storage. In contrast, *C. goreaui* displayed pronounced shifts in response to both Fe and N where under Fe limitation, chlorophyll and carbohydrate levels declined while Zn, Mn, and Cu quotas increased, indicative of possible metal substitution and compensatory regulation. Under high Fe, particularly with NH_4_^+^, *C. goreaui* accumulated lipids while carotenoid concentration decreased, reflecting a shift from photoprotection toward biosynthesis and carbon storage as energy and redox constraints eased. These observations suggest that while both species are constrained by Fe limitation, *S. microadriaticum* adopts a compensatory metabolic strategy to sustain photophysiology, whereas *C. goreaui* relies more heavily on trace metal homeostasis and resource reallocation. Collectively, these species-specific strategies highlight the diverse nutritional ecophysiology within Symbiodiniaceae and have implications for how distinct symbiont lineages may respond to nutrient variability in reef environments.

Overall, the synthesis of chlorophyll, carbohydrates, lipids, proteins, and carotenoids in Symbiodiniaceae is intricately linked to the availability of Fe, NO_3_^−^, NH_4_^+^ and other micronutrients in their environment. These nutrients not only support essential metabolic pathways but also regulate the physiological responses of dinoflagellates to environmental fluctuations, ultimately influencing their capacity to sustain symbiotic relationships with hosts and contribute to the health and resilience of holobionts. The different responses in metal quotas between *S. microadriaticum* and *C. goreaui* shows their species-specific adaptations to environmental stressors, including nutrient limitations. These adaptations are crucial for their survival and growth in marine ecosystems, particularly in nutrient-deficient regions or during seasonal fluctuations in nutrient availability. Unraveling these metal dynamics enhances our understanding of Symbiodiniaceae ecology and their contributions to global biogeochemical cycles, emphasizing the intricate interplay between trace metals and nutrient limitations in marine environments.

## Data Availability

The raw data supporting the conclusions of this article will be made available by the authors, without undue reservation.

## Supplementary material

SUPPLEMENTARY FIGURE S1 Maximum cell density **(A)** and P content **(B)** of *S. microadriaticum* and *C. goreaui* subjected to varying Fe and N treatments. Bars represent mean values with error bars showing standard deviations from triplicate culture bottles. Letters above bars indicate Tukey’s post-hoc groupings for significant differences.

SUPPLEMENTARY FIGURE S2 Intracellular Fe **(A)**, Zn **(B)**, Co **(C)**, Mn **(D)**, and Cu **(E)** concentration, in fmol cell^-1^, of *S. microadriaticum* and *C. goreaui* subjected to varying Fe and N treatments. Boxplots represent range of values with error bars showing standard deviations from triplicate culture bottles. Letters above boxplots indicate Tukey’s post-hoc groupings for significant differences. Asterisks show significant differences between species on the same treatments.
