# Supplementary figures and images for: Ammonium supply represses iron limitation to support Symbiodiniaceae growth
Source: Front Microbiol. 2025 Oct 28;16:1663314. doi: 10.3389/fmicb.2025.1663314 (PMC12602481; doi:10.3389/fmicb.2025.1663314)

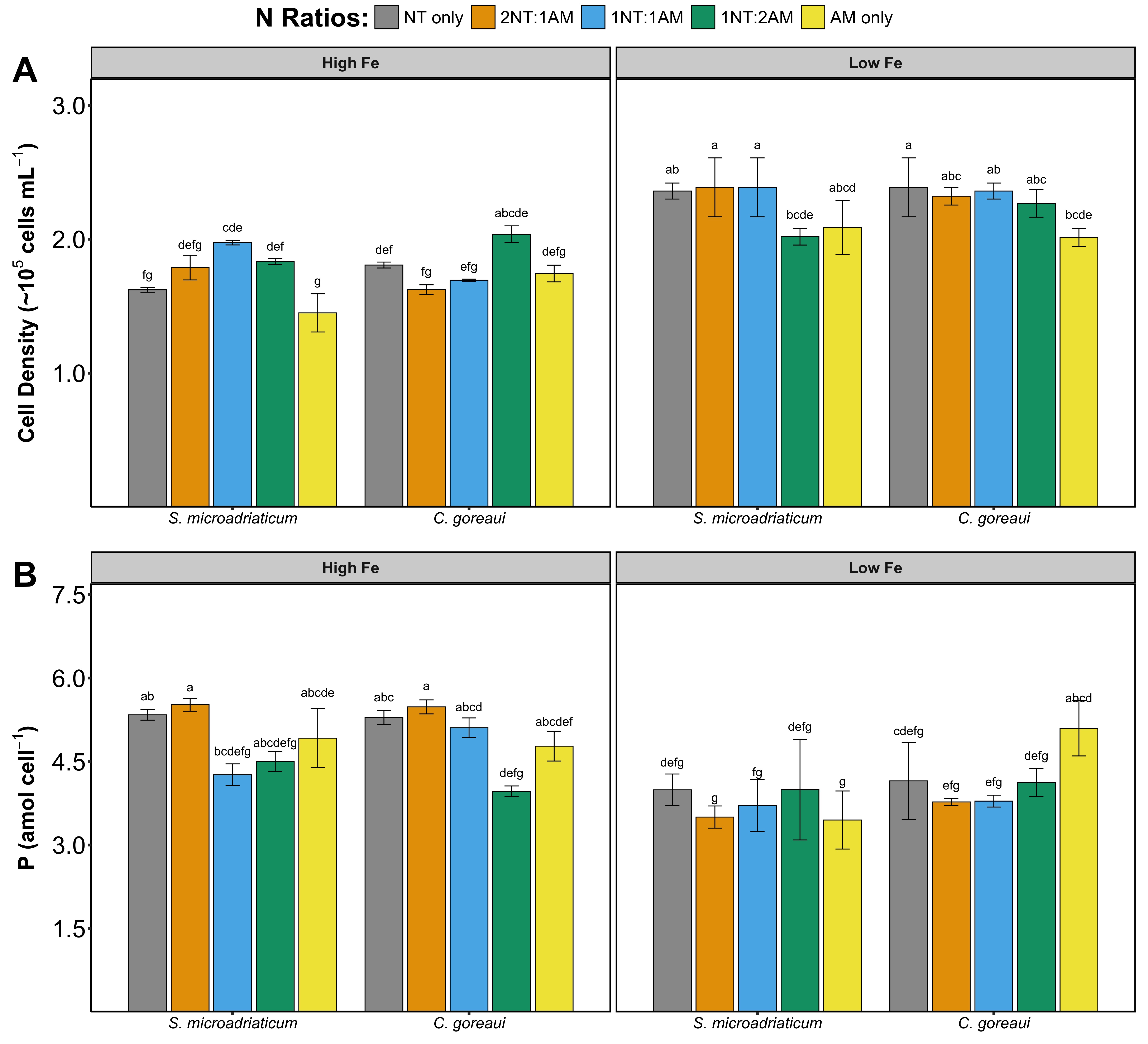

Supplement: SUPPLEMENTARY FIGURE S1 — Maximum cell density (A) and P content (B) of S. microadriaticum and C. goreaui subjected to varying Fe and N treatments. Bars represent mean values with error bars showing standard deviations from triplicate culture bottles. Letters above bars indicate Tukey’s post-hoc groupings for significant differences. [file Image_1.JPEG]

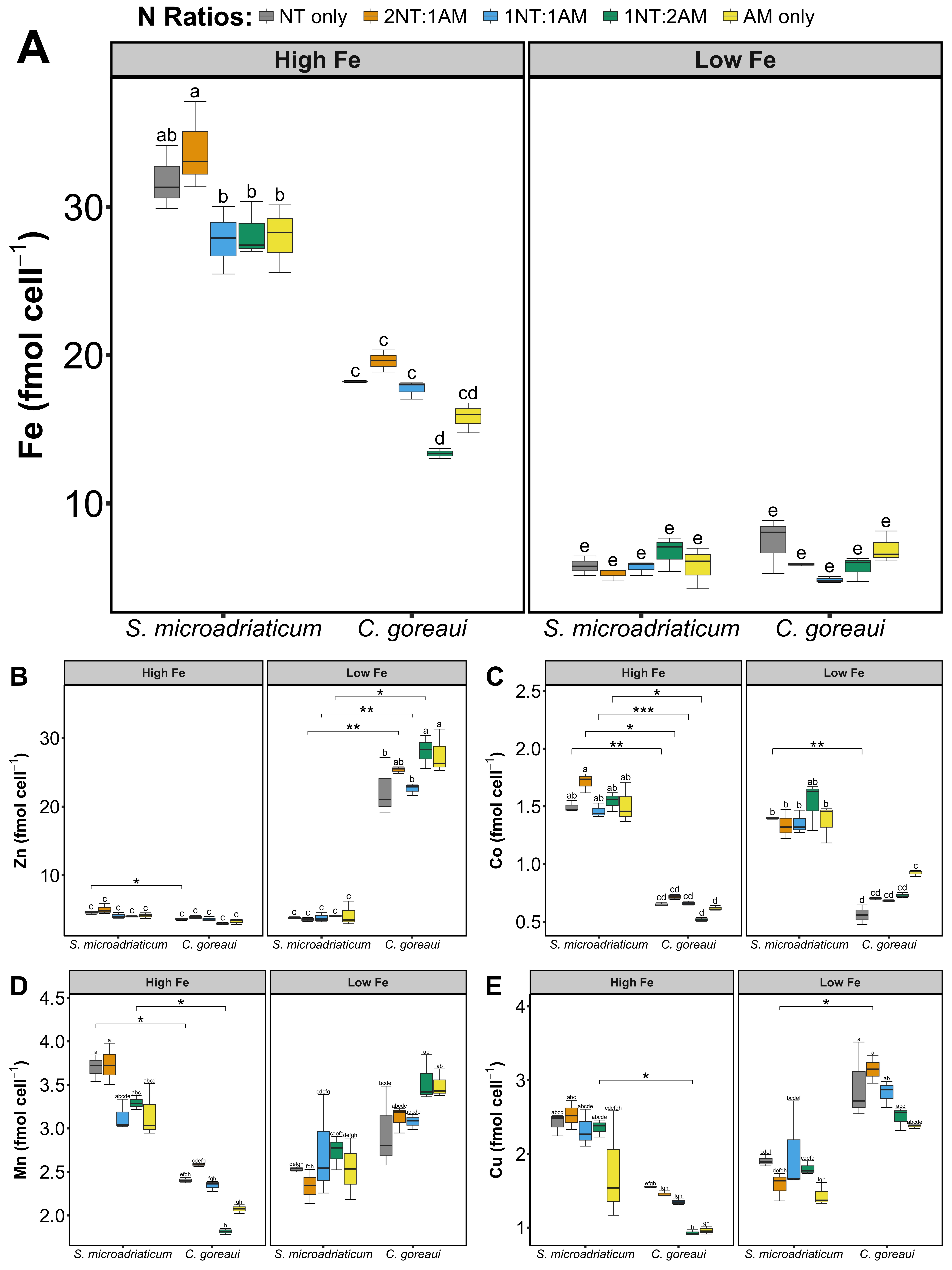

Supplement: SUPPLEMENTARY FIGURE S2 — Intracellular Fe (A), Zn (B), Co (C), Mn (D), and Cu (E) concentration, in fmol cell-1, of S. microadriaticum and C. goreaui subjected to varying Fe and N treatments. Boxplots represent range of values with error bars showing standard deviations from triplicate culture bottles. Letters above boxplots indicate Tukey’s post-hoc groupings for significant differences. Asterisks show significant differences between species on the same treatments. [file Image_2.JPEG]
