# Supplementary material for: Ammonium supply represses iron limitation to support Symbiodiniaceae growth
Source: Front Microbiol. 2025 Oct 28;16:1663314. doi: 10.3389/fmicb.2025.1663314 (PMC12602481; doi:10.3389/fmicb.2025.1663314)
Supplement: Supplementary file 3 [file Table_1.DOCX]

**Table S1.** Composition of modified L1 medium.

|  | **Primary Stock**  **(mol/L)** | **Working Stock**  **(mol/L)** | **Final Concentration in Seawater Medium**  **(mol/L)** |
| --- | --- | --- | --- |
| **Major Nutrients** | | | |
| NaH_2_PO_4_ • H_2_O |  | 1.25 × 10^-02^ | **1.25 × 10^-05^** |
| *NaNO_3_ |  | 2.00 × 10^-01^ | **2.00 × 10^-04^** |
| *NH_3_Cl |  | 2.00 × 10^-01^ | **2.00 × 10^-04^** |
| **Trace Metals** | | | |
| MnCl_2_ • 4H_2_O | 1.00 × 10^-02^ | 1.00 × 10^-05^ | **1.00 × 10^-08^** |
| ZnSO_4_ • 7H_2_O | 1.00 × 10^-02^ | 1.00 × 10^-05^ | **1.00 × 10^-08^** |
| CoCl_2_ •6H_2_O | 1.00 × 10^-02^ | 1.00 × 10^-05^ | **1.00 × 10^-08^** |
| Na_2_MoO_2_ • 2H_2_O | 1.00 × 10^-02^ | 1.00 × 10^-05^ | **1.00 × 10^-08^** |
| CuSO_4_ • 5H_2_O | 1.00 × 10^-02^ | 1.00 × 10^-05^ | **1.00 × 10^-08^** |
| Na_2_SeO4 | 1.00 × 10^-02^ | 1.00 × 10^-05^ | **1.00 × 10^-08^** |
| NiSO_4_ • 6H_2_O | 1.00 × 10^-01^ | 1.00 × 10^-05^ | **1.00 × 10^-08^** |
| *FeCl_3_ • 6H_2_O | 1.00 × 10^-01^ | 1.00 × 10^-04^ | **1.00 × 10^-07^** |
| **Vitamins** | | | |
| B12 | 3.96 × 10^-05^ | 3.96 × 10^-07^ | **3.96 × 10^-10^** |
| Biotin | 2.50 × 10^-04^ | 2.50 × 10^-06^ | **2.50 × 10^-09^** |
| Thiamine | 2.96 × 10^-02^ | 2.96 × 10^-04^ | **2.96 × 10^-07^** |
| **Others** | | | |
| Na_2_EDTA • 2H_2_O |  | 2.00 × 10^-02^ | **2.00 × 10^-05^** |
| ^1^ This version of the L1 medium by Guillard and Hargraves (1993) was based on experimental results of Rodriguez et al. (2016) on *Fugacium kawagutii* (formerly known as *Symbiodinium kawagutii*).  ^2^ Stock are made with ultrapure deionized water. Trace metal stocks are acidified up to 1 M of HCl to increase metal solubility to solvent.  ^3^ Filtered natural seawater was used as the base of the medium.  ^4^ NH_3_Cl was not introduced until preparation of experimental treatments.  ^5^ Silicate was not added because, as dinoflagellates, Symbiodiniaceae do not require it.  *Final concentrations of NO_3_^-^, NH_4_^+^, and Fe were dictated by experimental treatments | | | |
