# Supplementary material for: Ammonium supply represses iron limitation to support Symbiodiniaceae growth
Source: Front Microbiol. 2025 Oct 28;16:1663314. doi: 10.3389/fmicb.2025.1663314 (PMC12602481; doi:10.3389/fmicb.2025.1663314)
Supplement: Supplementary file 4 [file Table_2.DOCX]

**Table S2.** Summary of physiological responses of Symbiodiniaceae subjected to varying Fe and N availability.

| **Variables** | ***S. microadriaticum*** | | ***C. goreaui*** | |
| --- | --- | --- | --- | --- |
|  | **High Fe Conditions** | **Low Fe Conditions** | **High Fe Conditions** | **Low Fe Conditions** |
| **Growth** | | | | |
| **Specific Growth Rate** | no changes in NO_3_⁻  no changes in NH_4_^+^ | **↓** in NO_3_⁻  no changes in NH_4_^+^ | no changes in NO_3_⁻  no changes in NH_4_^+^ | **↓** in NO_3_⁻  **↓** in NH_4_^+^ |
| **Intracellular Metal Quotas** | | | | |
| **Fe** | no changes in NO_3_⁻  no changes in NH_4_^+^ | **↓** in NO_3_⁻  **↓** in NH_4_^+^ | no changes in NO_3_⁻  no changes in NH_4_^+^ | **↓** in NO_3_⁻  **↓** in NH_4_^+^ |
| **Zn** | no changes in NO_3_⁻  no changes in NH_4_^+^ | no changes in NO_3_⁻  **↑** in NH_4_^+^ | no changes in NO_3_⁻  no changes in NH_4_^+^ | no changes in NO_3_⁻  **↑** in NH_4_^+^ |
| **Co** | **↓** in NO_3_⁻  **↓** in NH_4_^+^ | no changes in NO_3_⁻  **↑** in NH_4_^+^ | no changes in NO_3_⁻  no changes in NH_4_^+^ | no changes in NO_3_⁻  no changes in NH_4_^+^ |
| **Mn** | no changes in NO_3_⁻  no changes in NH_4_^+^ | no changes in NO_3_⁻  no changes in NH_4_^+^ | no changes in NO_3_⁻  no changes in NH_4_^+^ | **↑** in NO_3_⁻  **↑** in NH_4_^+^ |
| **Cu** | no changes in NO_3_⁻  no changes in NH_4_^+^ | no changes in NO_3_⁻  no changes in NH_4_^+^ | no changes in NO_3_⁻  no changes in NH_4_^+^ | **↑** in NO_3_⁻  **↑** in NH_4_^+^ |
| **Pigments** | | | | |
| **Chlorophyll** | no changes in NO_3_⁻  **↑** in NH_4_^+^ | no changes in NO_3_⁻  no changes in NH_4_^+^ | **↓** in NO_3_⁻  **↑** in NH_4_^+^ | **↓** in NO_3_⁻  **↓** in NH_4_^+^ |
| **Carotenoids** | **↑** in NO_3_⁻  no changes in NH_4_^+^ | **↑** in NO_3_⁻  **↑** in NH_4_^+^ | **↑** in NO_3_⁻  no changes in NH_4_^+^ | **↑** in NO_3_⁻  **↑** in NH_4_^+^ |
| **Macromolecules** | | | | |
| **Carbohydrates** | no changes in NO_3_⁻  **↑** in NH_4_^+^ | **↓** in NO_3_⁻  **↓** in NH_4_^+^ | no changes in NO_3_⁻  **↑** in NH_4_^+^ | **↓** in NO_3_⁻  **↓** in NH_4_^+^ |
| **Lipids** | **↓** in NO_3_⁻  **↑** in NH_4_^+^ | **↓** in NO_3_⁻  no changes in NH_4_^+^ | no changes in NO_3_⁻  **↑** in NH_4_^+^ | **↓** in NO_3_⁻  no changes in NH_4_^+^ |
| **Proteins** | no changes in NO_3_⁻  no changes in NH_4_^+^ | no changes in NO_3_⁻  **↑** in NH_4_^+^ | no changes in NO_3_⁻  **↑** in NH_4_^+^ | no changes in NO_3_⁻  **↑** in NH_4_^+^ |

^1^Results are relative to HFe 1NT:1AM treatment per species.
