# Supplementary material for: Ammonium supply represses iron limitation to support Symbiodiniaceae growth
Source: Front Microbiol. 2025 Oct 28;16:1663314. doi: 10.3389/fmicb.2025.1663314 (PMC12602481; doi:10.3389/fmicb.2025.1663314)
Supplement: Supplementary file 5 [file Table_3.DOCX]

**Table S3.** Three-way analysis of variance (ANOVA) for specific growth rates of *S. microadriaticum* and *C. goreaui* subjected to varying Fe and N availability.

|  | **df** | **Sum of Squares** | **Mean of Squares** | **F-value** | **p-value** |
| --- | --- | --- | --- | --- | --- |
| Species | 1 | 0.025 | 0.249 | 532.18 | **< 2.00 × 10^-16^** |
| Fe Level | 1 | 0.039 | 0.393 | 840.80 | **< 2.00 × 10^-16^** |
| N Ratio | 4 | 0.002 | < 0.001 | 9.38 | **1.92 × 10^-5^** |
| Species : Fe Level | 1 | 0.014 | 0.014 | 301.64 | **< 2.00 × 10^-16^** |
| Species : N Ratio | 4 | 0.001 | < 0.001 | 3.73 | **0.011** |
| Fe Level : N Ratio | 4 | 0.023 | 0.001 | 12.72 | **9.09 × 10^-16^** |
| Species : Fe Level : N Ratio | 4 | 0.003 | 0.001 | 14.75 | **1.73 × 10^-7^** |
| Residuals | 40 | 0.002 | < 0.001 |  |  |

Species: S. microadriaticum vs. C. goreaui

Fe Level: High Fe vs Low Fe

N Ratio: NT-only vs. 2NT:1AM vs. 1NT:1AM vs. 1NT:2AM vs. AM-only

**Table S4.** Bonferroni-adjusted paired t-test results for specific growth rates of *S. microadriaticum* (i) and *C. goreaui* (j) subjected to varying Fe and N availability.

| **Fe Level** | **N Ratio** | **Mean Difference**  **(i-j)** | **Standard Error** | **Lower Limit** | **Upper Limit** | **p-value** |
| --- | --- | --- | --- | --- | --- | --- |
| High | NT-only | -0.081 | 0.005 | -0.153 | -0.008 | **0.040*** |
|  | 2NT:1AM | -0.039 | 0.005 | -0.108 | 0.031 | **0.157** |
|  | 1NT:1AM | -0.063 | 0.008 | -0.173 | 0.046 | **0.147** |
|  | 1NT:2AM* | -0.099 | 0.005 | -0.174 | -0.023 | **0.029** |
|  | AM-only | -0.075 | 0.007 | -0.180 | 0.030 | **0.096** |
| Low | NT-only | -0.010 | 0.007 | -0.111 | 0.092 | **1.000** |
|  | 2NT:1AM | -0.018 | 0.003 | -0.058 | 0.022 | **0.250** |
|  | 1NT:1AM | -0.015 | 0.008 | -0.121 | 0.091 | **1.000** |
|  | 1NT:2AM | 0.005 | 0.010 | -0.129 | 0.139 | **1.000** |
|  | AM-only | -0.013 | 0.005 | -0.087 | 0.060 | **1.000** |

Asterisks indicate significant differences between species on specific treatment/s.

**Table S5.** Three-way analysis of variance (ANOVA) for Fe quota of *S. microadriaticum* and *C. goreaui* subjected to varying Fe and N availability.

|  | **df** | **Sum of Squares** | **Mean of Squares** | **F-value** | **p-value** |
| --- | --- | --- | --- | --- | --- |
| Species | 1 | 28.97 | 28.97 | 679.51 | **< 2.00 × 10^-16^** |
| Fe Level | 1 | 159.09 | 159.09 | 3732.00 | **< 2.00 × 10^-16^** |
| N Ratio | 4 | 0.29 | 0.07 | 1.73 | **0.162** |
| Species : Fe Level | 1 | 24.95 | 24.95 | 585.24 | **< 2.00 × 10^-16^** |
| Species : N Ratio | 4 | 0.67 | 0.17 | 3.93 | **0.009** |
| Fe Level : N Ratio | 4 | 0.62 | 0.16 | 3.66 | **0.012** |
| Species : Fe Level : N Ratio | 4 | 0.25 | 0.06 | 1.47 | **0.230** |
| Residuals | 40 | 1.71 | 0.04 |  |  |

Species: S. microadriaticum vs. C. goreaui

Fe Level: High Fe vs Low Fe

N Ratio: NT-only vs. 2NT:1AM vs. 1NT:1AM vs. 1NT:2AM vs. AM-only

**Table S6.** Bonferroni-adjusted paired t-test results for Fe quota of *S. microadriaticum* (i) and *C. goreaui* (j) subjected to varying Fe and N availability.

| **Fe Level** | **N Ratio** | **Mean Difference**  **(i-j)** | **Standard Error** | **Lower Limit** | **Upper Limit** | **p-value** |
| --- | --- | --- | --- | --- | --- | --- |
| High | NT-only | 2.507 | 0.213 | -0.494 | 5.508 | **0.071** |
|  | 2NT:1AM | 2.553 | 0.357 | -2.470 | 7.578 | **0.190** |
|  | 1NT:1AM | 3.044 | 0.151 | 0.920 | 5.168 | **0.024** |
|  | 1NT:2AM | 2.907 | 0.217 | -0.153 | 5.966 | **0.055** |
|  | AM-only* | 2.386 | 0.112 | 0.783 | 3.988 | **0.023** |
| Low | NT-only | -0.304 | 0.034 | -0.779 | 0.171 | **0.121** |
|  | 2NT:1AM | -0.061 | 0.020 | -0.337 | 0.215 | **0.896** |
|  | 1NT:1AM | 0.255 | 0.095 | -1.078 | 1.588 | **1.000** |
|  | 1NT:2AM | 0.319 | 0.146 | -1.744 | 2.383 | **1.000** |
|  | AM-only | 0.291 | 0.196 | -2.464 | 3.046 | **1.000** |

Asterisks indicate significant differences between species on specific treatment/s.

**Table S7.** Three-way analysis of variance (ANOVA) for Zn quota of *S. microadriaticum* and *C. goreaui* subjected to varying Fe and N availability.

|  | **df** | **Sum of Squares** | **Mean of Squares** | **F-value** | **p-value** |
| --- | --- | --- | --- | --- | --- |
| Species | 1 | 88.40 | 88.40 | 629.19 | **< 2.00 × 10^-16^** |
| Fe Level | 1 | 115.27 | 115.27 | 820.45 | **< 2.00 × 10^-16^** |
| N Ratio | 4 | 1.40 | 0.35 | 2.50 | **0.058** |
| Species : Fe Level | 1 | 102.62 | 102.62 | 730.41 | **< 2.00 × 10^-16^** |
| Species : N Ratio | 4 | 1.40 | 0.35 | 2.48 | **0.059** |
| Fe Level : N Ratio | 4 | 1.07 | 0.27 | 1.91 | **0.129** |
| Species : Fe Level : N Ratio | 4 | 1.39 | 0.35 | 2.48 | **0.059** |
| Residuals | 40 | 5.62 | 0.14 |  |  |

Species: S. microadriaticum vs. C. goreaui

Fe Level: High Fe vs Low Fe

N Ratio: NT-only vs. 2NT:1AM vs. 1NT:1AM vs. 1NT:2AM vs. AM-only

**Table S8.** Bonferroni-adjusted paired t-test results for Zn quota of *S. microadriaticum* (i) and *C. goreaui* (j) subjected to varying Fe and N availability.

| **Fe Level** | **N Ratio** | **Mean Difference**  **(i-j)** | **Standard Error** | **Lower Limit** | **Upper Limit** | **p-value** |
| --- | --- | --- | --- | --- | --- | --- |
| High | NT-only* | 0.176 | 0.007 | 0.078 | 0.275 | **0.016** |
|  | 2NT:1AM | 0.202 | 0.076 | -0.875 | 1.279 | **1.000** |
|  | 1NT:1AM | 0.259 | 0.053 | -0.489 | 1.008 | **0.395** |
|  | 1NT:2AM | 0.144 | 0.016 | -0.088 | 0.376 | **0.128** |
|  | AM-only | 0.159 | 0.050 | -0.546 | 0.864 | **0.867** |
| Low | NT-only | -4.560 | 0.756 | -15.215 | 6.095 | **0.264** |
|  | 2NT:1AM** | -5.703 | 0.096 | -7.049 | -4.356 | **0.003** |
|  | 1NT:1AM** | -4.943 | 0.123 | -6.676 | -3.210 | **0.006** |
|  | 1NT:2AM* | -5.770 | 0.388 | -11.232 | -0.308 | **0.045** |
|  | AM-only | -4.240 | 0.562 | -12.164 | 3.683 | **0.171** |

Asterisks indicate significant differences between species on specific treatment/s.

**Table S9.** Three-way analysis of variance (ANOVA) for Co quota of *S. microadriaticum* and *C. goreaui* subjected to varying Fe and N availability.

|  | **df** | **Sum of Squares** | **Mean of Squares** | **F-value** | **p-value** |
| --- | --- | --- | --- | --- | --- |
| Species | 1 | 0.58 | 0.58 | 1743.42 | **< 2.00 × 10^-16^** |
| Fe Level | 1 | 0.04 | 0.04 | 124.95 | **7.07 × 10^-14^** |
| N Ratio | 4 | 0.01 | < 0.01 | 7.86 | **8.98 × 10^-5^** |
| Species : Fe Level | 1 | < 0.01 | < 0.01 | 3.23 | **0.080** |
| Species : N Ratio | 4 | < 0.01 | < 0.01 | 1.06 | **0.389** |
| Fe Level : N Ratio | 4 | < 0.01 | < 0.01 | 1.98 | **0.117** |
| Species : Fe Level : N Ratio | 4 | < 0.01 | < 0.01 | 2.38 | **0.068** |
| Residuals | 40 | 0.01 | < 0.01 |  |  |

Species: S. microadriaticum vs. C. goreaui

Fe Level: High Fe vs Low Fe

N Ratio: NT-only vs. 2NT:1AM vs. 1NT:1AM vs. 1NT:2AM vs. AM-only

**Table S10.** Bonferroni-adjusted paired t-test results for Co quota of *S. microadriaticum* (i) and *C. goreaui* (j) subjected to varying Fe and N availability.

| **Fe Level** | **N Ratio** | **Mean Difference**  **(i-j)** | **Standard Error** | **Lower Limit** | **Upper Limit** | **p-value** |
| --- | --- | --- | --- | --- | --- | --- |
| High | NT-only** | 0.158 | 0.002 | 0.125 | 0.191 | **0.002** |
|  | 2NT:1AM* | 0.179 | 0.007 | 0.083 | 0.275 | **0.014** |
|  | 1NT:1AM*** | 0.214 | 0.002 | 0.190 | 0.237 | **0.001** |
|  | 1NT:2AM* | 0.213 | 0.014 | 0.010 | 0.416 | **0.045** |
|  | AM-only** | 0.178 | 0.004 | 0.129 | 0.227 | **0.004** |
| Low | NT-only | 0.213 | 0.025 | -0.144 | 0.571 | **0.139** |
|  | 2NT:1AM* | 0.196 | 0.012 | 0.024 | 0.369 | **0.039** |
|  | 1NT:1AM | 0.187 | 0.018 | -0.071 | 0.445 | **0.095** |
|  | 1NT:2AM | 0.212 | 0.027 | -0.170 | 0.594 | **0.159** |
|  | AM-only* | 0.217 | 0.012 | 0.050 | 0.384 | **0.020** |

Asterisks indicate significant differences between species on specific treatment/s.

**Table S11.** Three-way analysis of variance (ANOVA) for Mn quota of *S. microadriaticum* and *C. goreaui* subjected to varying Fe and N availability.

|  | **df** | **Sum of Squares** | **Mean of Squares** | **F-value** | **p-value** |
| --- | --- | --- | --- | --- | --- |
| Species | 1 | 0.09 | 0.09 | 24.81 | **1.26 × 10^-5^** |
| Fe Level | 1 | 0.40 | 0.40 | 115.75 | **2.25 × 10^-13^** |
| N Ratio | 4 | 0.04 | 0.01 | 3.03 | **0.029** |
| Species : Fe Level | 1 | 0.41 | 0.41 | 117.36 | **1.83 × 10^-13^** |
| Species : N Ratio | 4 | 0.02 | < 0.01 | 1.35 | **0.268** |
| Fe Level : N Ratio | 4 | 0.01 | < 0.01 | 0.85 | **0.501** |
| Species : Fe Level : N Ratio | 4 | 0.03 | 0.01 | 2.11 | **0.097** |
| Residuals | 40 | 0.14 | < 0.01 |  |  |

Species: S. microadriaticum vs. C. goreaui

Fe Level: High Fe vs Low Fe

N Ratio: NT-only vs. 2NT:1AM vs. 1NT:1AM vs. 1NT:2AM vs. AM-only

**Table S12.** Bonferroni-adjusted paired t-test results for Mn quota of *S. microadriaticum* (i) and *C. goreaui* (j) subjected to varying Fe and N availability.

| **Fe Level** | **N Ratio** | **Mean Difference**  **(i-j)** | **Standard Error** | **Lower Limit** | **Upper Limit** | **p-value** |
| --- | --- | --- | --- | --- | --- | --- |
| High | NT-only* | 0.239 | 0.014 | 0.042 | 0.436 | **0.034** |
|  | 2NT:1AM | 0.204 | 0.018 | -0.055 | 0.464 | **0.080** |
|  | 1NT:1AM* | 0.276 | 0.010 | 0.135 | 0.417 | **0.013** |
|  | 1NT:2AM* | 0.274 | 0.018 | 0.017 | 0.530 | **0.044** |
|  | AM-only* | 0.209 | 0.015 | 0.001 | 0.417 | **0.050** |
| Low | NT-only | -0.091 | 0.090 | -1.359 | 1.178 | **1.000** |
|  | 2NT:1AM | -0.160 | 0.032 | -0.615 | 0.294 | **0.382** |
|  | 1NT:1AM | -0.080 | 0.053 | -0.830 | 0.670 | **1.000** |
|  | 1NT:2AM | -0.157 | 0.055 | -0.937 | 0.622 | **1.000** |
|  | AM-only | -0.043 | 0.072 | -0.975 | 1.062 | **1.000** |

Asterisks indicate significant differences between species on specific treatment/s.

**Table S13.** Three-way analysis of variance (ANOVA) for Cu quota of *S. microadriaticum* and *C. goreaui* subjected to varying Fe and N availability.

|  | **df** | **Sum of Squares** | **Mean of Squares** | **F-value** | **p-value** |
| --- | --- | --- | --- | --- | --- |
| Species | 1 | < 0.01 | < 0.01 | 0.09 | **0.766** |
| Fe Level | 1 | 0.68 | 0.68 | 139.10 | **1.35 × 10^-14^** |
| N Ratio | 4 | 0.19 | 0.05 | 9.70 | **1.35 × 10^-5^** |
| Species : Fe Level | 1 | 0.66 | 0.66 | 135.81 | **1.96 × 10^-14^** |
| Species : N Ratio | 4 | 0.06 | 0.02 | 3.14 | **0.025** |
| Fe Level : N Ratio | 4 | 0.03 | 0.01 | 1.68 | **0.173** |
| Species : Fe Level : N Ratio | 4 | 0.06 | 0.01 | 2.87 | **0.035** |
| Residuals | 40 | 0.20 | < 0.01 |  |  |

Species: S. microadriaticum vs. C. goreaui

Fe Level: High Fe vs Low Fe

N Ratio: NT-only vs. 2NT:1AM vs. 1NT:1AM vs. 1NT:2AM vs. AM-only

**Table S14.** Bonferroni-adjusted paired t-test results for Cu quota of *S. microadriaticum* (i) and *C. goreaui* (j) subjected to varying Fe and N availability.

| **Fe Level** | **N Ratio** | **Mean Difference**  **(i-j)** | **Standard Error** | **Lower Limit** | **Upper Limit** | **p-value** |
| --- | --- | --- | --- | --- | --- | --- |
| High | NT-only | 0.159 | 0.017 | -0.080 | 0.398 | **0.112** |
|  | 2NT:1AM | 0.192 | 0.017 | -0.054 | 0.438 | **0.082** |
|  | 1NT:1AM* | 0.281 | 0.019 | 0.015 | 0.547 | **0.045** |
|  | 1NT:2AM | 0.289 | 0.023 | -0.036 | 0.614 | **0.063** |
|  | AM-only | 0.158 | 0.089 | -1.094 | 1.410 | **1.000** |
| Low | NT-only | -0.239 | 0.091 | -1.527 | 1.049 | **1.000** |
|  | 2NT:1AM | -0.385 | 0.031 | -0.815 | 0.045 | **0.062** |
|  | 1NT:1AM | -0.207 | 0.085 | -1.404 | 0.989 | **1.000** |
|  | 1NT:2AM | -0.143 | 0.022 | -0.452 | 0.166 | **0.227** |
|  | AM-only | -0.050 | 0.049 | -0.743 | 0.643 | **1.000** |

Asterisks indicate significant differences between species on specific treatment/s.

**Table S15.** Three-way analysis of variance (ANOVA) for chlorophyll content of *S. microadriaticum* and *C. goreaui* subjected to varying Fe and N availability.

|  | **df** | **Sum of Squares** | **Mean of Squares** | **F-value** | **p-value** |
| --- | --- | --- | --- | --- | --- |
| Species | 1 | 0.01 | 0.01 | 0.64 | **0.429** |
| Fe Level | 1 | 0.80 | 0.80 | 48.13 | **2.29 × 10^-8^** |
| N Ratio | 4 | 0.95 | 0.24 | 14.27 | **2.55 × 10^-7^** |
| Species : Fe Level | 1 | 0.04 | 0.04 | 2.50 | **0.122** |
| Species : N Ratio | 4 | 0.03 | 0.01 | 0.42 | **0.791** |
| Fe Level : N Ratio | 4 | 0.33 | 0.08 | 4.88 | **0.003** |
| Species : Fe Level : N Ratio | 4 | 0.01 | < 0.01 | 0.08 | **0.988** |
| Residuals | 40 | 0.67 | 0.02 |  |  |

Species: S. microadriaticum vs. C. goreaui

Fe Level: High Fe vs Low Fe

N Ratio: NT-only vs. 2NT:1AM vs. 1NT:1AM vs. 1NT:2AM vs. AM-only

**Table S16.** Bonferroni-adjusted paired t-test results for chlorophyll content of *S. microadriaticum* (i) and *C. goreaui* (j) subjected to varying Fe and N availability.

| **Fe Level** | **N Ratio** | **Mean Difference**  **(i-j)** | **Standard Error** | **Lower Limit** | **Upper Limit** | **p-value** |
| --- | --- | --- | --- | --- | --- | --- |
| High | NT-only | -0.057 | 0.147 | -2.125 | 2.011 | **1.000** |
|  | 2NT:1AM | -0.130 | 0.065 | -1.047 | 0.787 | **1.000** |
|  | 1NT:1AM | 3.70 × 10^-17^ | 0.050 | -0.709 | 0.709 | **1.000** |
|  | 1NT:2AM | 0.433 | 0.529 | -0.695 | 0.781 | **1.000** |
|  | AM-only | 0.013 | 0.104 | -1.452 | 1.478 | **1.000** |
| Low | NT-only | 0.067 | 0.156 | -2.138 | 2.271 | **1.000** |
|  | 2NT:1AM | 0.033 | 0.095 | -1.304 | 1.371 | **1.000** |
|  | 1NT:1AM | 0.110 | 0.026 | -0.263 | 0.483 | **0.533** |
|  | 1NT:2AM | 0.097 | 0.144 | -1.933 | 2.126 | **1.000** |
|  | AM-only | 0.090 | 0.100 | -1.322 | 1.501 | **1.000** |

Asterisks indicate significant differences between species on specific treatment/s.

**Table S17.** Three-way analysis of variance (ANOVA) for carotenoid content of *S. microadriaticum* and *C. goreaui* subjected to varying Fe and N availability.

|  | **df** | **Sum of Squares** | **Mean of Squares** | **F-value** | **p-value** |
| --- | --- | --- | --- | --- | --- |
| Species | 1 | 0.41 | 0.41 | 29.04 | **3.40 × 10^-6^** |
| Fe Level | 1 | 2.29 | 2.29 | 163.46 | **1.03 × 10^-15^** |
| N Ratio | 4 | 1.01 | 0.25 | 17.95 | **1.64 × 10^-8^** |
| Species : Fe Level | 1 | 0.21 | 0.21 | 15.25 | **< 0.001** |
| Species : N Ratio | 4 | 0.10 | 0.03 | 1.83 | **0.143** |
| Fe Level : N Ratio | 4 | 0.08 | 0.02 | 1.48 | **0.225** |
| Species : Fe Level : N Ratio | 4 | 0.01 | < 0.01 | 0.16 | **0.956** |
| Residuals | 40 | 0.56 | 0.01 |  |  |

Species: S. microadriaticum vs. C. goreaui

Fe Level: High Fe vs Low Fe

N Ratio: NT-only vs. 2NT:1AM vs. 1NT:1AM vs. 1NT:2AM vs. AM-only

**Table S18.** Bonferroni-adjusted paired t-test results for carotenoid content of *S. microadriaticum* (i) and *C. goreaui* (j) subjected to varying Fe and N availability.

| **Fe Level** | **N Ratio** | **Mean Difference**  **(i-j)** | **Standard Error** | **Lower Limit** | **Upper Limit** | **p-value** |
| --- | --- | --- | --- | --- | --- | --- |
| High | NT-only | -0.213 | 0.054 | -0.969 | 0.542 | **0.578** |
|  | 2NT:1AM | -0.010 | 0.029 | -0.417 | 0.397 | **1.000** |
|  | 1NT:1AM | -0.003 | 0.043 | -0.614 | 0.607 | **1.000** |
|  | 1NT:2AM | -0.040 | 0.085 | -1.238 | 1.158 | **1.000** |
|  | AM-only | 0.040 | 0.055 | -0.736 | 0.816 | **1.000** |
| Low | NT-only | -0.427 | 0.084 | -1.606 | 0.753 | **0.364** |
|  | 2NT:1AM | -0.180 | 0.076 | -1.247 | 0.887 | **1.000** |
|  | 1NT:1AM | -0.227 | 0.104 | -1.691 | 1.238 | **1.000** |
|  | 1NT:2AM | -0.317 | 0.096 | -1.669 | 1.036 | **0.809** |
|  | AM-only | -0.270 | 0.180 | -2.807 | 2.267 | **1.000** |

Asterisks indicate significant differences between species on specific treatment/s.

**Table S19.** Three-way analysis of variance (ANOVA) for chlorophyll/carotenoid of *S. microadriaticum* and *C. goreaui* subjected to varying Fe and N availability.

|  | **df** | **Sum of Squares** | **Mean of Squares** | **F-value** | **p-value** |
| --- | --- | --- | --- | --- | --- |
| Species | 1 | 0.08 | 0.08 | 3.57 | **0.066** |
| Fe Level | 1 | 2.57 | 2.57 | 121.23 | **1.12 × 10^-13^** |
| N Ratio | 4 | 2.46 | 0.61 | 29.00 | **2.39 × 10^-11^** |
| Species : Fe Level | 1 | 0.09 | 0.09 | 4.06 | **0.051** |
| Species : N Ratio | 4 | 0.04 | 0.01 | 0.52 | **0.725** |
| Fe Level : N Ratio | 4 | 1.05 | 0.26 | 12.38 | **1.21 × 10^-6^** |
| Species : Fe Level : N Ratio | 4 | 0.01 | < 0.01 | 0.17 | **0.955** |
| Residuals | 40 | 0.85 | 0.02 |  |  |

Species: S. microadriaticum vs. C. goreaui

Fe Level: High Fe vs Low Fe

N Ratio: NT-only vs. 2NT:1AM vs. 1NT:1AM vs. 1NT:2AM vs. AM-only

**Table S20.** Bonferroni-adjusted paired t-test results for chlorophyll/carotenoid of *S. microadriaticum* (i) and *C. goreaui* (j) subjected to varying Fe and N availability.

| **Fe Level** | **N Ratio** | **Mean Difference**  **(i-j)** | **Standard Error** | **Lower Limit** | **Upper Limit** | **p-value** |
| --- | --- | --- | --- | --- | --- | --- |
| High | NT-only | 0.060 | 0.111 | -1.498 | 1.618 | **1.000** |
|  | 2NT:1AM | -0.107 | 0.073 | -1.137 | 0.923 | **1.000** |
|  | 1NT:1AM | 3.701 × 10^-17^ | 0.025 | -0.355 | 0.355 | **1.000** |
|  | 1NT:2AM | 0.090 | 0.095 | -1.254 | 1.434 | **1.000** |
|  | AM-only | -0.067 | 0.228 | -3.282 | 3.149 | **1.000** |
| Low | NT-only | 0.117 | 0.088 | -1.118 | 1.351 | **1.000** |
|  | 2NT:1AM | 0.077 | 0.072 | -0.940 | 1.094 | **1.000** |
|  | 1NT:1AM | 0.153 | 0.043 | -0.446 | 0.753 | **0.691** |
|  | 1NT:2AM | 0.213 | 0.153 | -1.949 | 2.375 | **1.000** |
|  | AM-only | 0.173 | 0.148 | -1.907 | 2.254 | **1.000** |

Asterisks indicate significant differences between species on specific treatment/s.

**Table S21.** Three-way analysis of variance (ANOVA) for carbohydrate content of *S. microadriaticum* and *C. goreaui* subjected to varying Fe and N availability.

|  | **df** | **Sum of Squares** | **Mean of Squares** | **F-value** | **p-value** |
| --- | --- | --- | --- | --- | --- |
| Species | 1 | 132 | 132 | 2.09 | **0.156** |
| Fe Level | 1 | 22110 | 22110 | 350.23 | **< 2.00 × 10^-16^** |
| N Ratio | 4 | 5994 | 1499 | 23.74 | **4.13 × 10^-10^** |
| Species : Fe Level | 1 | 22 | 22 | 0.35 | **0.559** |
| Species : N Ratio | 4 | 59 | 15 | 0.23 | **0.919** |
| Fe Level : N Ratio | 4 | 853 | 213 | 0.38 | **0.018** |
| Species : Fe Level : N Ratio | 4 | 34 | 9 | 0.14 | **0.968** |
| Residuals | 40 | 2525 | 63 |  |  |

Species: S. microadriaticum vs. C. goreaui

Fe Level: High Fe vs Low Fe

N Ratio: NT-only vs. 2NT:1AM vs. 1NT:1AM vs. 1NT:2AM vs. AM-only

**Table S22.** Bonferroni-adjusted paired t-test results for carbohydrate content of *S. microadriaticum* (i) and *C. goreaui* (j) subjected to varying Fe and N availability.

| **Fe Level** | **N Ratio** | **Mean Difference**  **(i-j)** | **Standard Error** | **Lower Limit** | **Upper Limit** | **p-value** |
| --- | --- | --- | --- | --- | --- | --- |
| High | NT-only | -4.313 | 15.296 | -219.819 | 211.192 | **1.000** |
|  | 2NT:1AM | 1.337 | 13.693 | -191.578 | 194.251 | **1.000** |
|  | 1NT:1AM | 2.100 | 3.803 | -51.480 | 55.680 | **1.000** |
|  | 1NT:2AM | 4.150 | 6.938 | -93.606 | 101.906 | **1.000** |
|  | AM-only | 5.500 | 9.377 | -126.610 | 137.610 | **1.000** |
| Low | NT-only | 2.613 | 2.166 | -27.903 | 33.130 | **1.000** |
|  | 2NT:1AM | 5.753 | 4.071 | -51.604 | 63.111 | **1.000** |
|  | 1NT:1AM | 4.347 | 7.998 | -108.340 | 117.033 | **1.000** |
|  | 1NT:2AM | 4.207 | 2.794 | -35.164 | 43.577 | **1.000** |
|  | AM-only | 3.957 | 2.690 | -33.941 | 41.854 | **1.000** |

Asterisks indicate significant differences between species on specific treatment/s.

**Table S23.** Three-way analysis of variance (ANOVA) for lipid content of *S. microadriaticum* and *C. goreaui* subjected to varying Fe and N availability.

|  | **df** | **Sum of Squares** | **Mean of Squares** | **F-value** | **p-value** |
| --- | --- | --- | --- | --- | --- |
| Species | 1 | 364382 | 364382 | 1202.65 | **< 2.00 × 10^-16^** |
| Fe Level | 1 | 97694 | 97694 | 322.44 | **< 2.00 × 10^-16^** |
| N Ratio | 4 | 108961 | 27240 | 8991 | **< 2.00 × 10^-16^** |
| Species : Fe Level | 1 | 39885 | 39885 | 131.64 | **3.17 × 10^-14^** |
| Species : N Ratio | 4 | 17012 | 4253 | 14.04 | **3.06 × 10^-7^** |
| Fe Level : N Ratio | 4 | 10509 | 2627 | 8.67 | **3.88 × 10^-5^** |
| Species : Fe Level : N Ratio | 4 | 1238 | 309 | 1.02 | **0.408** |
| Residuals | 40 | 12119 | 303 |  |  |

Species: S. microadriaticum vs. C. goreaui

Fe Level: High Fe vs Low Fe

N Ratio: NT-only vs. 2NT:1AM vs. 1NT:1AM vs. 1NT:2AM vs. AM-only

**Table S24.** Bonferroni-adjusted paired t-test results for lipid content of *S. microadriaticum* (i) and *C. goreaui* (j) subjected to varying Fe and N availability.

| **Fe Level** | **N Ratio** | **Mean Difference**  **(i-j)** | **Standard Error** | **Lower Limit** | **Upper Limit** | **p-value** |
| --- | --- | --- | --- | --- | --- | --- |
| High | NT-only** | -213.213 | 5.194 | -285.547 | -140.879 | **0.006** |
|  | 2NT:1AM** | -201.017 | 5.406 | -277.176 | -124.858 | **0.007** |
|  | 1NT:1AM | -162.277 | 21.052 | -458.877 | 134.324 | **0.164** |
|  | 1NT:2AM | -176.733 | 16.180 | -404.694 | 51.227 | **0.083** |
|  | AM-only | -283.883 | 33.771 | -759.682 | 191.916 | **0.139** |
| Low | NT-only* | -110.560 | 4.897 | -179.549 | -41.571 | **0.020** |
|  | 2NT:1AM | -90.467 | 9.011 | -217.423 | 36.489 | **0.098** |
|  | 1NT:1AM | -84.207 | 6.183 | -171.318 | 2.905 | **0.053** |
|  | 1NT:2AM | -84.580 | 19.723 | -362.465 | 193.305 | **0.503** |
|  | AM-only** | -151.657 | 4.281 | -211.969 | -91.345 | **0.008** |

Asterisks indicate significant differences between species on specific treatment/s.

**Table S25.** Three-way analysis of variance (ANOVA) for protein content of *S. microadriaticum* and *C. goreaui* subjected to varying Fe and N availability.

|  | **df** | **Sum of Squares** | **Mean of Squares** | **F-value** | **p-value** |
| --- | --- | --- | --- | --- | --- |
| Species | 1 | 39111 | 39111 | 34.08 | **7.99 × 10^-7^** |
| Fe Level | 1 | 39478 | 39478 | 34.40 | **7.31 × 10^-7^** |
| N Ratio | 4 | 121651 | 30413 | 26.50 | **8.80 × 10^-11^** |
| Species : Fe Level | 1 | 2411 | 2411 | 2.10 | **0.155** |
| Species : N Ratio | 4 | 15827 | 3957 | 3.45 | **0.016** |
| Fe Level : N Ratio | 4 | 16746 | 4187 | 3.65 | **0.013** |
| Species : Fe Level : N Ratio | 4 | 3115 | 779 | 0.68 | **0.611** |
| Residuals | 40 | 45902 | 1148 |  |  |

Species: S. microadriaticum vs. C. goreaui

Fe Level: High Fe vs Low Fe

N Ratio: NT-only vs. 2NT:1AM vs. 1NT:1AM vs. 1NT:2AM vs. AM-only

**Table S26.** Bonferroni-adjusted paired t-test results for protein content of *S. microadriaticum* (i) and *C. goreaui* (j) subjected to varying Fe and N availability.

| **Fe Level** | **N Ratio** | **Mean Difference**  **(i-j)** | **Standard Error** | **Lower Limit** | **Upper Limit** | **p-value** |
| --- | --- | --- | --- | --- | --- | --- |
| High | NT-only | 98.840 | 21.024 | -197.375 | 395.055 | **0.424** |
|  | 2NT:1AM | 92.387 | 10.925 | -61.536 | 246.309 | **0.137** |
|  | 1NT:1AM | 93.273 | 16.482 | -138.938 | 325.485 | **0.298** |
|  | 1NT:2AM | 4.820 | 10.337 | -140.818 | 150.458 | **1.000** |
|  | AM-only | 29.390 | 39.293 | -524.211 | 582.991 | **1.000** |
| Low | NT-only* | 83.730 | 1.296 | 65.465 | 101.995 | **0.002** |
|  | 2NT:1AM | 62.733 | 24.511 | -282.602 | 408.068 | **1.000** |
|  | 1NT:1AM | 26.783 | 23.682 | -307.083 | 360.650 | **1.000** |
|  | 1NT:2AM | 26.373 | 33.373 | -443.815 | 496.562 | **1.000** |
|  | AM-only | -7.703 | 32.061 | -459.411 | 444.004 | **1.000** |

Asterisks indicate significant differences between species on specific treatment/s.
